# Evaluation of the relationship between Hoffa volume and radiological and clinical scoring in the diagnosis and treatment of anterior knee pain: A retrospective observational study

**DOI:** 10.1097/MD.0000000000043244

**Published:** 2025-07-04

**Authors:** Siddik Göksel Gürsoy, Ahmet Emrah Açan, Serdar Sargin, Özgür Baykan, Sercan Tosun, Erdoğan Bülbül

**Affiliations:** a Department of Orthopedics and Traumatology, Faculty of Medicine, Balikesir University, Balikesir, Turkey; b Department of Biochemistry, Faculty of Medicine, Balikesir University, Balikesir, Turkey; c Department of Radiology, Faculty of Medicine, Balikesir University, Balikesir, Turkey.

**Keywords:** anterior knee pain, Hoffa disease, Hoffa syndrome, impingement, volume

## Abstract

This study aimed to evaluate the relationship between clinical outcomes and Hoffa fat pad (HFP) volume with respect to treatment type: conventional therapy (NSAIDs and topical cold) and intra-articular platelet-rich plasma (PRP). This retrospective study included patients aged 18 to 40 years with anterior knee pain (AKP), positive Hoffa squeeze and extension impingement tests, and no radiological pathology. After applying the inclusion and exclusion criteria, the patients were divided into conventional treatment (n = 28) and PRP (n = 27) groups, with a control group (n = 25) to compare the baseline HFP volume. HFP volume was measured pre- and posttreatment using 3D Slicer software, while clinical outcomes were assessed using Lysholm, Oxford Knee, and visual analog scale (VAS) scores. Baseline HFP volumes did not differ significantly between the groups (*P* = .236). The conventional group showed a significant reduction in HFP volume, from 30.8 ± 5.9 cm^3^ to 29.5 ± 5.7 cm^3^ (*P* = .002), while the PRP group saw an increase from 28.0 ± 5.3 cm^3^ to 29.9 ± 6.3 cm^3^ (*P* = .001). Baseline VAS scores were higher in the PRP group (7.1 ± 0.9 vs 6.0 ± 1.0, *P* < .001), with posttreatment VAS scores improving in both groups (PRP: 3.0, *P* < .001; Conventional: 1.5, *P* < .001). Lysholm and Oxford scores increased significantly posttreatment (*P* < .001), although no correlation was found between HFP volume and these scores. These findings suggest Hoffa volume may influence AKP, although volume changes do not directly correlate with functional improvement, indicating that other pathophysiological factors may play a role. These findings highlight the need for further studies to understand HFP’s role of HFP in AKP and inform targeted treatments.

## 1. Introduction

Anterior knee pain (AKP) is a common complaint, especially among young adults and athletes, and can significantly limit physical activity and reduce the quality of life.^[1]^ AKP is a complex syndrome with multiple etiologies, including patellofemoral pain syndrome, patellar tendinopathy, and synovitis, yet AKP cases without identifiable radiological pathology often remain undiagnosed.^[2]^ This creates a diagnostic and therapeutic challenge, as symptoms may persist or worsen if untreated. Among the various anatomical structures within the knee, Hoffa fat pad (HFP), also known as the infrapatellar fat pad, is increasingly recognized as a potential contributor to undiagnosed AKP and is called Hoffa fat pat impingement.^[3]^

The HFP is an intracapsular and extrasynovial tissue located between the patellar tendon and tibia. It is rich in vascularization and innervation by the posterior articular branch of the tibial nerve that courses through the external borders of the menisci, synovium, and cruciate ligaments. Owing to its flexibility and displaceability, it can accommodate different degrees of flexion-extension of the knee, serving as in joint lubrication and cushioning.^[3]^ Recently, HFP has been considered another regulator of joint inflammation by secreting multiple endocrine factors and inflammatory cytokines similar to the synovia and a reservoir for multipotent cells.^[4,5]^ The dimensions, configuration including clefts, and volume of this fat pad vary widely among individuals.^[6–8]^

Hoffa impingement was first described by Albert Hoffa in 1904 as an inflammatory fibrous hyperplasia of the infrapatellar fat pad, resulting in symptomatic relief after excision in a series of 21 patients.^[9]^ It was further classified as primary hypertrophy and secondary hypertrophy, which is more prevalent and often associated with other pathologies such as meniscal tears and ligament injuries. More recently, the primary type, associated with edema and fibrosis in a normal joint, has been referred to as Hoffa disease, whereas the secondary type is called Hoffa syndrome.^[10]^ In both types, the mechanism involves hemorrhage, inflammation, and hypertrophy of the HFP in the acute phase, followed by fibrosis as the condition becomes chronic owing to repetitive microtrauma or coexisting knee pathologies, eventually leading to impingement at the tibiofemoral joint and the lateral aspect of the patellofemoral joint.

Diagnosis of Hoffa disease is challenging. It presents with a prolonged period of AKP, typically in the infrapatellar or retropatellar tendon region, which is exacerbated by movement or loading of the extended knee or prolonged flexion, and is often confused with meniscal and patellar pathologies.^[11]^ However, there are currently no pathognomonic radiological findings to definitively diagnose HFP-related AKP.^[3,10–13]^ As a result, diagnosis often relies more on clinical examinations than on radiological approaches, using tests such as the Hoffa squeeze test and the extension impingement test, while excluding other AKP pathologies.^[12]^ Additionally, the relationship between HFP volume and AKP symptoms remains underexplored, missing the cutoff volume value to define Hoffa hypertrophy.^[12]^ Additionally, changes in HFP volume over time, as well as its relationship to pain, functional impairment, and type of treatment have not been reported in the literature.^[10,14]^

Considering the literature indicating that Hoffa disease arises from fat pad impingement, our hypothesis is that the success of treatment will correlate with the reduction in HFP volume.^[14]^ The primary aim of this study was to evaluate the relationship between clinical and functional outcomes and HFP volume in relation to the type of treatment: conventional treatment (CoT), including non-steroidal anti-inflammatory drugs (NSAIDs) and topical cold application, and alternative treatment with intra-articular platelet-rich plasma (PRP) injection. The secondary aim of this study was to compare the HFP volume of the control group with that of the treatment groups.

## 2. Methods

This retrospective cohort study was conducted with ethical approval from the institutional review board (approval number 2023/161), ensuring adherence to ethical standards. The inclusion criteria were as follows: patients aged 18 to 40 years presenting with AKP and no knee pathology detected on initial MRI at presentation; positive results on both the Hoffa test and the extension impingement test; treatment with either conventional therapy (NSAIDs and topical cold application) or intra-articular PRP injection; a follow-up MRI conducted 6 months posttreatment; completion of both pretreatment and posttreatment clinical and functional outcome assessments; adherence to treatment protocols and consistent attendance at follow-up visits; and sufficient cognitive ability to complete surveys, along with a willingness to participate in the study. The exclusion criteria were as follows: a history of surgery, major trauma, septic arthritis, or osteoarthritis affecting the knee joint or adjacent joints (hip and ankle); a diagnosis of rheumatologic disease, neurological disease, or radiculopathy; lower limb deformities; radiological findings of intra-articular pathologies such as patellofemoral, meniscal, chondral, or tendinous pathologies that could cause AKP; a history of patellar instability; evidence of patellofemoral maltracking, patella alta, or patella baja; and incomplete follow-up.

Participants were divided into 3 groups. Group 1 was the control group, which consisted of asymptomatic individuals with lumbar pain but no reported knee pathologies. Group 2 and group 3 were based on the treatment they received: CoT and PRP Treatment, respectively. All patients were examined by the same surgeon and provided with 2 treatment options. Patients who opted for PRP treatment and those who chose conventional therapy were classified into separate groups, with consistent protocols followed within each group. Both treatment groups were advised to rest, including avoiding activities that aggravate pain such as deep knee flexion or high-impact sports. Patients in the CoT group received 90 mg of an oral NSAID (acemetacin) daily for 20 days, along with twice-daily topical cold application for 20 min and topical NSAID (ibuprofen) gel application. The aim of this treatment is to reduce inflammation and alleviate pain through conservative management. The PRP group received 2 PRP injections, 14 days apart. PRP was prepared by drawing 20 cc of blood, mixed with 3 cc of sodium citrate as an anticoagulant, and centrifuged twice at 1200 g for 5 min to yield 5 cc of PRP (Easy PRP KIT, Neotec Biotechnology, Turkey). PRP was injected intra-articularly by the same surgeon using the superolateral patellar approach. The rationale for using PRP stems from its potential to stimulate tissue repair and reduce inflammation through growth factor release.^[15]^ Each PRP injection was administered under aseptic conditions by the same orthopedic specialist.

Both groups underwent retrospective baseline assessments, including demographic data such as age, sex, and body mass index (BMI), as well as detailed physical examinations of the lower limb and radiological imaging (radiographs, orthoroentgenogram, and MRI) performed by the same orthopedic surgeon to apply the inclusion and exclusion criteria. Additionally, clinical and functional outcomes were retrospectively evaluated by another author using the Lysholm, Oxford, and visual analogue scale (VAS) scores.^[16,17]^ All these evaluations were performed at baseline and again 6 to 8 months posttreatment to determine changes in parameters.

**Figure 1.**
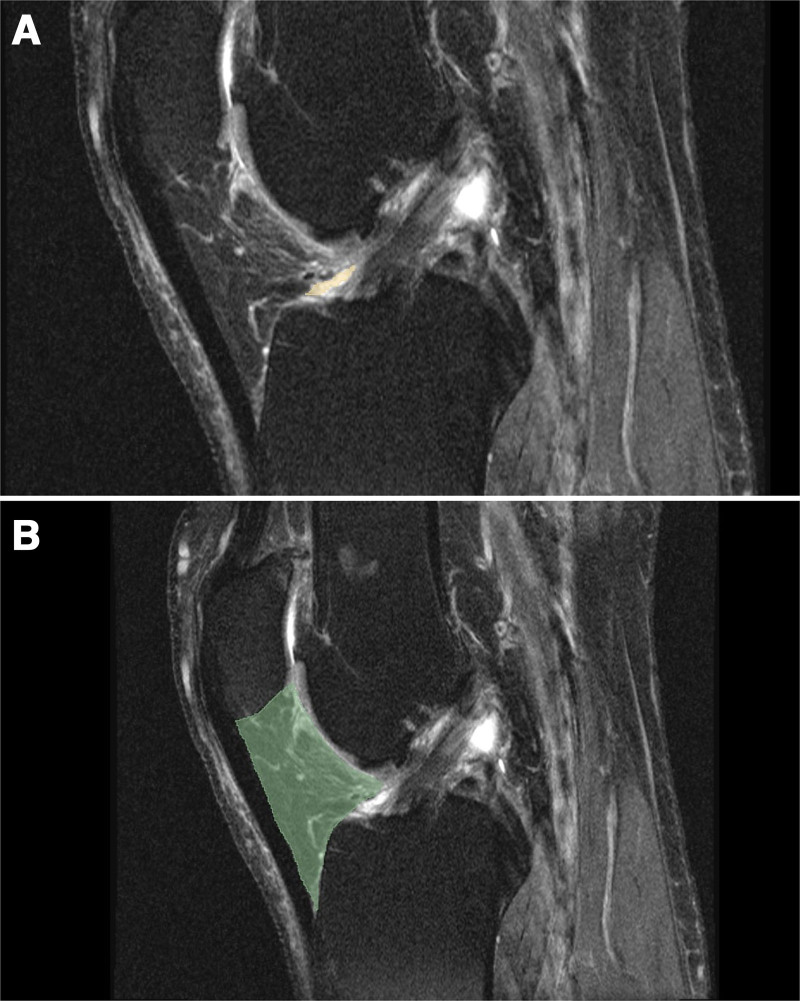
(A) Sagittal MRI section of a patient. (B) The boundaries of Hoffa fat pad in the sagittal MRI section were identified through segmentation using the 3D Slicer Software. MRI = magnetic resonance imaging.

The HFP volume was measured on MRI before and after treatment using an open-source software 3D Slicer version 5.6.2 (https://www.slicer.org/), which enabled precise segmentation and volumetric analysis of MRI scans. The HFP region was segmented manually, and volume measurements were calculated in cubic millimeters (mm³) (Fig. 1). These measurements were performed by the same radiologist, who was blinded to the groups.

### 2.1. Statistical analysis

Statistical analyses were performed using SPSS 25.0 (IBM, Chicago). Normality of data distribution was assessed using visual graphs (e.g., histograms) and statistical tests (Kolmogorov–Smirnov and Shapiro–Wilk). For normally distributed data, independent samples were compared using Student *t* test, while paired samples were analyzed using the paired *t* test. For non-normally distributed data, the Mann–Whitney U test was used for independent samples, and the Wilcoxon signed-rank test was used for paired samples. For comparisons involving more than 2 independent groups, ANOVA was applied for normally distributed data, followed by Tukey test for *post hoc* comparisons. For non-normally distributed data, the Kruskal–Wallis test was used, with the Mann–Whitney U test and Bonferroni correction applied for *post hoc* comparisons when applicable. Pearson or Spearman correlation analysis was used to evaluate the relationship between continuous variables, based on the normality of the data distribution. Categorical data were analyzed using Chi-square tests through cross-tabulations, and statistical significance was set at *P* < .05.

## 3. Results

Of the 111 patients initially evaluated for inclusion in the treatment groups, 24 were excluded due to additional pathologies detected on MRI. Four did not attend follow-up appointments, 7 lacked a second MRI, 2 experienced trauma during follow-up, 11 could not be contacted, 4 changed their physician, one required additional anti-inflammatory medication due to concurrent pathologies, and 3 underwent surgery during the follow-up period. Following the implementation of the inclusion and exclusion criteria, the treatment groups comprised 55 patients (22 females and 33 males). In addition, 25 individuals (10 females and 15 males) with no knee joint complaints and no pathologies on MRI were included in the control group. In conclusion, 80 patients (32 female and 48 male) were included in the study: 25 in the control group, 28 in the CoT group, and 27 in the PRP group. No statistically significant differences were observed between the groups in terms of age (*P* = .714), sex (*P* = .212), or BMI (*P* = .872) distribution (Table 1). Additionally, no statistically significant differences were found in the patients’ BMI between the baseline and post treatment values, either within groups (*P* = .285 for the PRP group, *P* = .309 for the CoT group).

**Table 1:** The age, gender, and BMI distribution of patients in each group.

Group	N	Age + SD	Female	Male	BMI + SD pretreatment	BMI + SD posttreatment
CoT	28	30.2 ± 5.3	8	20	24.7 ± 2.6	24.8 ± 2.6
PRP	27	29.5 ± 6.6	14	13	24.7 ± 2.9	24.6 ± 2.9
Control	25	28.9 ± 5.3	10	15	24.4 ± 3.1	–

BMI = body mass index, CoT = conventional treatment, PRP = platelet-rich plasma, SD = standard deviation.

In terms of baseline HFP volume measurements, the control group had a mean of 29.1 ± 7.3 cm^3^ (range: 15.8 to 41.3). In the PRP group, the mean volume was 28.0 ± 5.3 cm^3^ (range: 16.3 to 35.8). In the CoT group, the mean volume was 30.8 ± 5.9 cm^3^ (range: 20.6 to 43.2). No statistically significant difference was observed between the groups in terms of the baseline HFP volume (*P* = .236).

Following treatment, the mean posttreatment HFP volume was 29.9 ± 6.3 cm^3^ (range: 17.8 to 43.2) in the PRP group. In the CoT group, the mean volume was 29.5 ± 5.7 cm^3^ (range: 19.5 to 41.5). No statistically significant difference was found between the 2 groups in terms of the posttreatment HFP volume (*P* = .797). However, when the baseline and posttreatment HFP volumes were compared within the treatment groups, a statistically significant difference was found in both groups (*P* = .002 for the CoT group and *P* = .001 for the PRP group) (Table 2).

**Table 2:** The distribution of Hoffa fat pad volume in each group.

Group	Hoffa fat pad volume (cm^3^)				*P*-value
	Pretreatment		Posttreatment		
	Mean ± SD	Range	Mean ± SD	Range	
CoT	30.8 ± 5.9	20.6–43.2	29.5 ± 5.7	19.5–41.5	.002
PRP	28.0 ± 5.3	16.3–35.8	29.9 ± 6.3	17.8–43.2	.001
Control	29.1 ± 7.3	15.8–41.3			

CoT = conventional treatment, PRP = platelet-rich plasma, SD = standard deviation.

In terms of baseline VAS scores, the PRP group had a mean score of 7.1 ± 0.9 (range: 5 to 9), while the CoT group had a mean score of 6.0 ± 1.0 (range: 4 to 8). A statistically significant difference was found between the groups in terms of pretreatment VAS scores (*P* < .001). Regarding posttreatment VAS scores, the PRP group had a mean of 4.1 ± 1.8 (range: 1 to 8), and the CoT group had a mean of 4.5 ± 1.7 (range: 2 to 8). No statistically significant difference was observed between the groups in terms of the posttreatment VAS scores (*P* = .366). When comparing pre- and posttreatment VAS scores within each group to assess treatment effectiveness, the decrease in VAS score was an average of 3.0 in the PRP group and 1.5 in the CoT group was statistically significant (*P* < .001 for both groups) (Table 3).

**Table 3:** The distribution of pretreatment and posttreatment visual analog scale (VAS), Oxford, and Lysholm scores across each group.

Group	VAS (pretreatment) Mean ± SD	VAS (posttreatment) Mean ± SD	*P*-value
CoT	6.0 ± 1.0	4.5 ± 1.7	<.001
PRP	7.1 ± 0.9	4.1 ± 1.7	<.001

	Oxford Score (pretreatment) Mean + SD	Oxford Score (posttreatment) Mean + SD	
CoT	36.1 ± 2.8	37.4 ± 3.7	.005
PRP	35.1 ± 4.5	37.4 ± 5.0	<.001

	Lysholm Score (pretreatment) Mean + SD	Lysholm Score (posttreatment) Mean + SD	
CoT	69.5 ± 9.0	74.5 ± 11.1	.003
PRP	66.6 ± 9.9	78.2 ± 12.3	<.001

CoT = conventional treatment, PRP = platelet-rich plasma, SD = standard deviation, VAS = visual analog scale.

The pretreatment Lysholm scores of the patients were, on average, 66.6 ± 9.9 in the PRP group and 69.5 ± 9.0 in the CoT group, while posttreatment scores were 78.2 ± 12.3 and 74.5 ± 11.1, respectively. The increase in Lysholm score posttreatment was an average of 11.7 in the PRP group and 5.0 in the CoT group (*P* < .001 and *P* = .003, respectively). The pretreatment Oxford scores of the patients were, on average, 35.11 ± 4.492 in the PRP group and 36.07 ± 2.841 in the CoT group, while posttreatment Oxford scores were 37.41 ± 4.971 and 37.43 ± 3.686, respectively. The increase in Oxford score was an average of 2.30 in the PRP group and 1.36 in the CoT group (*P* < .001 and *P* = .005, respectively) (Table 3).

The HFP decreased from 30.8 cm^3^ to 29.5 cm^3^ in the CoT group, while the VAS scores also dropped from 6.0 to 4.5. However, no statistically significant correlation was observed between HFP volume and VAS scores after treatment (*r* = −0.282, *P* = .146). Meanwhile, in the PRP group, HFP volume increased from 20.8 cm^3^ to 29.9 cm^3^, while VAS scores decreased from 7.1 to 4.1. A statistically significant negative correlation was observed between HFP volume and VAS scores after treatment (*r* = −0.392, *P* = .043). The Lysolm (*P* = .278) and Oxford (*P* = .087) scores obtained after CoT showed no statistically significant relationship with HFP volume, and similarly, no statistically significant correlation was observed between HFP volume and Lysolm (*P* = .084) Oxford (*P* = .546) test results after PRP treatment.

## 4. Discussion

This study is the first to evaluate HFP volume changes both pre- and posttreatment in patients with AKP lacking a specific radiological pathology and to explore how these changes relate to clinical outcomes. Unlike previous studies that primarily considered HFP volume as a static measure, our study provides a dynamic perspective by assessing volume modifications following 2 different treatment modalities. This novel approach not only highlights the potential of HFP volume as a marker in AKP diagnosis, but also suggests that therapeutic interventions may influence its volume in ways that correlate with pain relief and functional improvements. Previous studies assessing the changes in HFP volume over time have investigated the effects of age and weight on HFP volume. Ladenhauf et al^[18]^ suggested that age might influence HFP volume, with an approximate annual increase of 2 cm³ in individuals aged between 4 and 17 years. In contrast, Chuckpaiwong et al^[19]^ in their adult cohort study, found no significant correlation between age and HFP volume. From the perspective of weight change, Steidle-Kloc et al,^[20]^ in their 2-year observational study, reported that the HFP volume decreased with weight loss, whereas no significant change was observed with weight gain. Similarly, Murillo et al^[21]^ reported a reduction in the HFP volume associated with weight loss. On the basis of these findings, we evaluated the pre- and posttreatment BMI values of the patients. No statistically significant differences were found in BMI between the baseline and posttreatment values, indicating that BMI changes did not interfere with the results of the HFP changes across groups.

The most notable and interesting finding in our study was that, while CoT with NSAIDs and cold application resulted in a decrease in HFP volume, PRP treatment led to significant clinical improvements without reducing HFP volume. The volume reduction in the CoT group aligns with the anti-inflammatory effects of NSAIDs, which inhibit cyclooxygenase and reduce prostaglandin synthesis, thereby mitigating inflammation within the HFP and alleviating symptoms. Additionally, cold application contributed to decreasing inflammation further by reducing local blood flow. However, the increase in HFP volume despite better outcomes with PRP contradicts our hypothesis that a greater reduction in HFP volume would lead to better clinical results, depending on the treatment method used. This finding does not support the hypertrophy of the HFP that cause inflammation and edema in the acute phase, as first described by Albert Hoffa in 1904.^[9]^ Although MRI offers valuable insights into HFP volume, it may not be sufficient as a standalone tool for treatment selection, given its static nature. In dynamic conditions such as Hoffa disease, volumetric MRI findings should be interpreted alongside clinical tests, such as the Hoffa squeeze and impingement maneuvers, to guide clinical decision-making. This suggests that despite over a century since its initial description, there may be additional overlooked pathogenic factors beyond HFP volume that contribute to the condition.

A plausible explanation for this paradox lies in the dual immunoregulatory role of the infrapatellar fat pad (HFP). As shown in previous studies, HFP can exhibit both pro-inflammatory and anti-inflammatory properties.^[4,5]^ We hypothesize that PRP may exert its clinical efficacy by promoting a shift toward an anti-inflammatory phenotype within the HFP. The volumetric increase observed may reflect increased metabolic or immunologic activity rather than edema or fibrotic hypertrophy. This hypothesis is supported by Jiang et al^[5]^ who proposed that intra-articular PRP may stimulate anti-inflammatory cascades within joint adipose tissue.

Hoffa disease is the primary type, whereas Hoffa syndrome is secondary and associated with various intra-articular pathologies, such as meniscopathy, patellar tendinopathies, or maltracking.^[10]^ Kumar et al^[22]^ reported a prevalence of 1.3% for the primary type and 6.8% for the secondary type among 2623 patients. The primary type with isolated HFP hypertrophy is diffucult to diagnose. These patients were generally in their twenties to thirties and presented clinically with AKP in the parapatellar or retropatellar region, which was aggravated by physical loading, particularly with full extension or prolonged knee flexion, and without any radiologically reported pathological findings.^[23]^ In clinical practice, MRI is the gold standard radiological imaging for knee joint injuries. However, there were no pathognomonic MRI findings.^[11]^ von Engelhardt et al^[3]^ analyzed preoperative MRIs findings in 62 patients with clinically suspected arthroscopically confirmed secondary infrapatellar fat pad impingement. Although no pathognomonic features were identified, they observed significantly higher rates of certain findings compared to the controls without impingement. These included edema of the superior/posterior fat pad (48% vs 27%) and an inflamed infrapatellar bursa (66% in impingement cases vs 43% in controls). Furthermore, there are no defined dimensions or cutoff values in the literature to categorize HFP volume as hypertrophic.^[3,6]^ Similarly, no statistically significant difference in HFP volume was observed between the control and treatment groups in our study. On the other hand, HFP impingement is a dynamic process that can be assessed by using high-resolution ultrasound to evaluate fat pad kinesiology as a non-invasive method rather than MRI as a static evaluation.^[10]^ Therefore, the diagnosis of HFP impingement is primarily based on clinical examination using the squeeze test and extension impingement test combined with radiological findings, while excluding other potential causes of AKP through both clinical and radiological evaluations.

The isolated type, known as Hoffa disease, lacks pathognomonic radiologic findings and is identified in only 1% of patients who have undergone arthroscopic surgery, leaving its true prevalence and percentage of patients requiring surgical intervention unclear.^[22,24]^ Additionally, due to limited literature, a standardized treatment algorithm for this condition has not yet been established. Conservative treatments include NSAIDs, cold application, rest, and avoidance of activities that exacerbate pain, such as deep knee flexion or high-impact sports. Physical therapy aimed at strengthening the vastus medialis obliquus muscle, patellar tendon offloading, HFP taping, weight loss, and manual therapy is also recommended.^[10,25]^ Furthermore, alcohol injection for therapeutic ablation and steroid injections for pain relief have been reported in the literature.^[10,26]^ However, these injections can be risky because of the close proximity of HFP to the patellar tendon.^[27]^ In addition, stem cell therapies using mesenchymal stem cells derived from the infrapatellar fat pad have shown potential in animal models to reverse synovitis and fibrosis of HFP.^[28,29]^ However, this is an expensive treatment, and further studies are necessary before they can be used in clinical practice. Intra-articular injection of PRP is widely used for various pathologies, including osteoarthritis and patellar chondromalacia.^[30]^ However, its use in Hoffa disease has not been reported in the literature. In our study, better outcomes were achieved with PRP; however, the reduction in HFP volume contrasts with the pathogenesis of HFP impingement, which is typically associated with HFP hypertrophy. This finding suggests the presence of overlooked immunopathological processes beyond just HFP volume, as HFP has been recognized as a regulator of joint inflammation, releasing hormones and inflammatory substances similar to the synovium.^[4,5]^ We believe that the better outcomes with PRP were the result of its modulatory role in inflammation and unknown crosstalk with HFP. As suggested by Jiang et al,^[5]^ HFP play a dual role, exhibiting both pro- and anti-inflammatory effects. In this context, we propose that intra-articular PRP, through an unknown crosstalk mechanism, stimulates HFP to function in a more anti-inflammatory manner, resulting in an increase in HFP volume due to its enhanced activity.

The primary limitations of this study are its relatively small patient population, relatively short follow-up period, and retrospective design, which introduces potential for selection bias. Another key limitation of this study was the absence of long-term clinical and radiological data on PRP treatment, leaving us uncertain whether the observed increase in HFP volume posttreatment might eventually decrease. Baseline VAS scores were notably higher in the PRP group than in the conventional group, which may suggest that patients experiencing more intense pain either leaned toward or were gently directed toward PRP treatment, indicating a potential selection bias as a limitation of the study. Additionally, if immunohistochemical analyses of samples obtained from HFP and knee joint fluid had been performed, they could have provided deeper insights into the underlying pathophysiology. Another critical limitation lies in the treatment group selection, as treatment choices were left to patient preference rather than randomized assignment. This introduces the risk of selection bias. Future randomized controlled trials are necessary to control for this bias and validate the findings in a more robust manner. Furthermore, while the dose itself was standardized, variability in the number of doses or timing between doses (e.g., administering one or 3 doses instead of 2 or extending the interval between doses from 2 to 3 weeks) was not explored, which could have influenced the outcomes and is a limitation of this study.

In conclusion, this study provides insights into the relationship between HFP volume changes and clinical outcomes in patients with AKP without specific radiological pathology. Our findings indicate that while conventional NSAID and cold application reduced HFP volume, PRP treatment improved clinical outcomes without reducing volume, suggesting a potential modulatory role of PRP in inflammation. However, further studies with larger populations, extended follow-up periods, randomized prospective designs, and immunohistochemical analyses are essential to better understand the mechanisms involved and to establish standardized treatment protocols for Hoffa disease.

## Author contributions

**Conceptualization:** Ahmet Emrah Açan, Serdar Sargin.

**Data curation:** Siddik Göksel Gürsoy, Özgür Baykan.

**Formal analysis:** Özgür Baykan.

**Investigation:** Siddik Göksel Gürsoy.

**Methodology:** Siddik Göksel Gürsoy, Ahmet Emrah Açan.

**Project administration:** Serdar Sargin.

**Software:** Sercan Tosun, Erdoğan Bülbül.

**Supervision:** Erdoğan Bülbül.

**Writing – original draft:** Ahmet Emrah Açan, Özgür Baykan.
